# Low-Cost Motility Tracking System (LOCOMOTIS) for Time-Lapse Microscopy Applications and Cell Visualisation

**DOI:** 10.1371/journal.pone.0103547

**Published:** 2014-08-14

**Authors:** Adam E. Lynch, Junian Triajianto, Edwin Routledge

**Affiliations:** 1 Institute for the Environment, Brunel University, Uxbridge, London, United Kingdom; 2 Karang Menjangan, Surabaya, Indonesia; University of Palermo, Italy

## Abstract

Direct visualisation of cells for the purpose of studying their motility has typically required expensive microscopy equipment. However, recent advances in digital sensors mean that it is now possible to image cells for a fraction of the price of a standard microscope. Along with low-cost imaging there has also been a large increase in the availability of high quality, open-source analysis programs. In this study we describe the development and performance of an expandable cell motility system employing inexpensive, commercially available digital USB microscopes to image various cell types using time-lapse and perform tracking assays in proof-of-concept experiments. With this system we were able to measure and record three separate assays simultaneously on one personal computer using identical microscopes, and obtained tracking results comparable in quality to those from other studies that used standard, more expensive, equipment. The microscopes used in our system were capable of a maximum magnification of 413.6×. Although resolution was lower than that of a standard inverted microscope we found this difference to be indistinguishable at the magnification chosen for cell tracking experiments (206.8×). In preliminary cell culture experiments using our system, velocities (mean µm/min ± SE) of 0.81±0.01 (*Biomphalaria glabrata* hemocytes on uncoated plates), 1.17±0.004 (MDA-MB-231 breast cancer cells), 1.24±0.006 (SC5 mouse Sertoli cells) and 2.21±0.01 (*B. glabrata* hemocytes on Poly-L-Lysine coated plates), were measured and are consistent with previous reports. We believe that this system, coupled with open-source analysis software, demonstrates that higher throughput time-lapse imaging of cells for the purpose of studying motility can be an affordable option for all researchers.

## Introduction

Cell motility has become an integrated measure used in a variety of modern assays spanning many research disciplines. Motile cells, individually or as groups, are vital to biological processes including fertilisation, growth and differentiation, immunity and the progression of diseases, such as cancer [1]–[3]. Consequently, a number of systems for studying motility are available to researchers and practitioners. For example, Trans-well assays (such as the Boyden chamber) enable measurements of cell motility in response to a chemical stimulus (chemotaxis). Chemotactic responses are quantified by the extent to which cells will migrate across a porous membrane towards a chosen test chemical [4]. However, despite being a relatively inexpensive means of measuring motility Trans-well assays do not permit direct observation of cells as they move [4], [5].

Direct visualisation is considered to be the ‘gold-standard’ in motility studies, enabling precise and continuous measurements of the speed, trajectory and morphology of individual cells under a microscope [6]. Cell motility is recorded by equipping the microscope with a digital camera and acquiring pictures at specific intervals over a chosen period of time (time-lapse) [1]. However, individual experiments often take several hours, and possibly days, to complete making these a daunting and laborious task when only a single microscope is available.

Over the last couple of decades, there have been considerable technological advancements in microscopy hardware and software to enable a large degree of automation over time-lapse studies [7], [8]. These advances include motorised stages and auto-focusing software which together allow the acquisition of images across numerous samples without the need for user intervention [8]. A basic commercial setup for performing motility studies usually requires an inverted microscope, digital camera, software and an incubator/heated stage and would be expected to cost several thousand pounds (GBP) whereas many automated systems can reach several hundred thousand pounds [9], [10]. Due to high software and hardware costs such systems are accessible only to those with large budgets, and are often outside the reach of non-specialist researchers, or those in developing countries with fewer resources [7]. Indeed, affordable solutions for the direct visualisation of cells is rapidly becoming a research area in its own right [11]–[14].

The recent ability to produce low-cost imaging devices is a consequence of improvements in image sensors such as charge coupled devices (CCDs) and complementary metal oxide semiconductors (CMOS) enabling good quality imaging together with substantial decreases in size and cost [13]. Such devices are now produced for a range of applications from disease diagnosis [12], [14] to measurement of sperm motility [15]. However, although these can vastly increase the affordability of motility assays and deliver high image quality, none can match the high-throughput nature of the more expensive devices. Therefore, the next stage in low-cost imaging will be to develop solutions to increase their throughput ability.

Along with advances in hardware, recent years have also seen a significant increase in the amount of high quality open-source software (which in many cases is comparable in capability to commercial packages) written for the analysis of microscopy data [16]. Here we show that by combining a number of low-cost imaging devices with open-source software we are entering a stage where high-throughput digital microscopy imaging can be an affordable option for all researchers. Building on existing work in low-cost imaging, we hope that this system will help make motility measurements accessible to all researchers, and inspire others to continue to enhance the productivity of similar devices.

## Materials and Methods

### Construction

**Figure 1:**
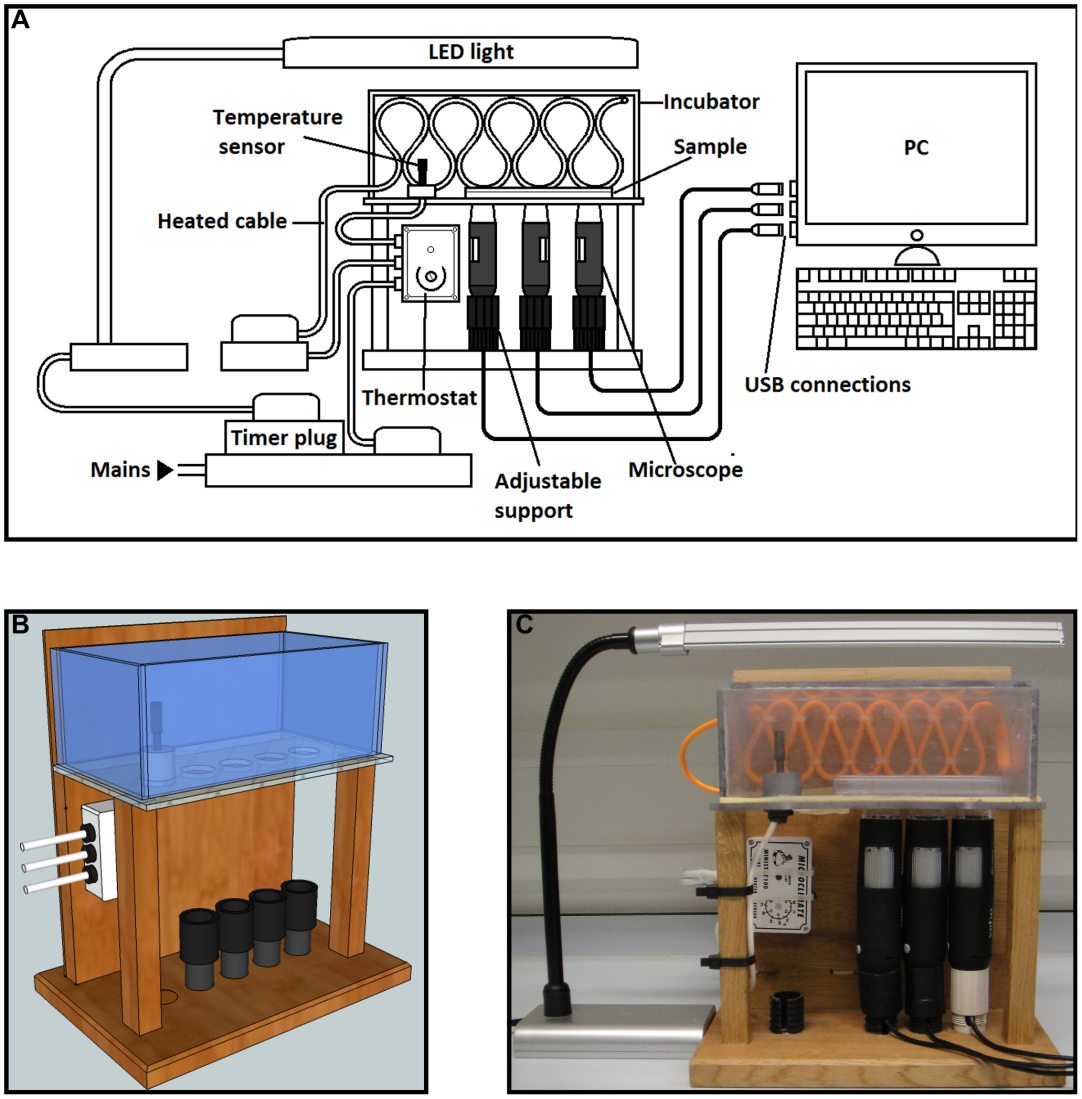
Images of the microscope system. (a) Schematic diagram of microscope system; (b) CAD model; (c) Photograph of finished system.

The design process (Figure 1A) began by creating a 3D model of the system in Trimble Sketchup 8 (Figure 1B). The frame was constructed using oak, the stage from acrylic and the microscope support bases were created from adjustable kitchen unit legs, which fit them well and allow the height to be adjusted so that the top of the microscope is level with the stage and secure which prevents image drift and allows for optimum magnification (Figure 1C). Detailed information regarding the construction process can be found in supporting information (Protocol S1, Protocol S2, Figure S1, Figure S2 and Table S1).

### Microscopes

All three microscopes used were identical models (VMS-004D, Veho, Hampshire, UK) in order to prevent any discrepancies. These microscopes use a CMOS image sensor with 1.3 mega-pixel resolution (2 MP with interpolation, although interpolation was not used in our studies). Magnification has two set levels (∼20× minimum and ∼400× maximum) achieved using a focusing wheel. To enhance stability, magnification and to allow for observation of live samples in liquid (cells) the microscopes were inverted.

### Lighting

Various lighting sources were tested, and ultimately an LED strip desk lamp was selected. The microscopes’ inbuilt LEDs were turned off. LEDs were used due to their low heat emission and intensity which helps reduce stress to the cells. To further prevent the risk of phototoxicity an accurate timer plug was used to turn the light off between image capturing. It was important to have a light source which could be adjusted since different samples have different requirements (due to depth or transparency).

### Heating

In order to keep samples at a constant physiological temperature, without needing to place the whole system inside an incubator, an incubation chamber was developed to fit over the top of the stage. The chamber was made from transparent acrylic to allow visualisation inside. The edges of the chamber were fitted with foam to improve insulation and the chamber was secured above the stage using metal clips. The heating element used was a 37.5 w soil warming cable; this was chosen since it is economical, waterproof and highly flexible. To maintain a constant temperature the heating cable was connected to a commercial mini thermostat of the kind typically used to heat vivariums (MicroClimate Ministat 100). The stability of the incubator temperature was measured at 27°C by placing a ‘Tinytag’ data logger (Gemini Data Loggers, Chichester, West Sussex, UK) inside which was programmed to take temperature measurements every minute for 20 hours.

### Resolution

The ultimate definition of resolution is described as the minimum distance two objects can be separated by and still are distinguished as separate features; this is sometimes referred to as spatial resolution. Spatial resolution is therefore the most important quantitative measurement. It is important to note that in digital systems spatial resolution is also related to, but not dependent on, pixel resolution which refers to the number of pixels utilised to create the image (width×height). In the case of digital imaging systems it is therefore also necessary to consider pixel resolution, as this can determine the amount of information from the real object that is retained within the digital image. If the number of pixels used to create the image is too low information is lost [18] [19].

Pixel resolution in Autokams was set at 640×480. Although the microscopes are capable of higher resolution, time-lapse studies typically generate a large number of files. Therefore, given the fact that three cameras were running together, a lower pixel resolution was chosen to reduce memory demand and enhance system stability. Spatial resolution was tested using 10 µl of a 0.1% solution of latex beads with a mean particle size of 1 µm (Sigma-Aldrich) trapped between two cover-slips.

### Magnification and Horizontal field of view

The calculation of digital image magnification is dependent on a number of factors and differs to those used in optical microscopy due to the lack of eye pieces and the fact that objects are viewed on a screen. Determining the magnification of an object on a screen is dependent on knowing the PPI (pixels per inch) of the screen, the size of a single pixel on the screen, the width of the image in pixels and the actual size of the object being magnified (Figure S3). Pixel resolution is also an important factor in determining ‘useful’ magnification as, up to a point, more pixels mean that the image contains more information which will be observable to the eye when it is enlarged. Simply put, magnification is used to make viewing easier, but if information is not already in the image increased magnification will not show any more detail [18].

Horizontal field of view (HFOV) and full magnification were measured using a calibration slide with a 1 mm marker and 10 µm subdivisions. The lower magnification and HFOV were measured with a 15 cm ruler with 1 mm subdivisions.

### Software

#### Image capture

**Figure 2:**
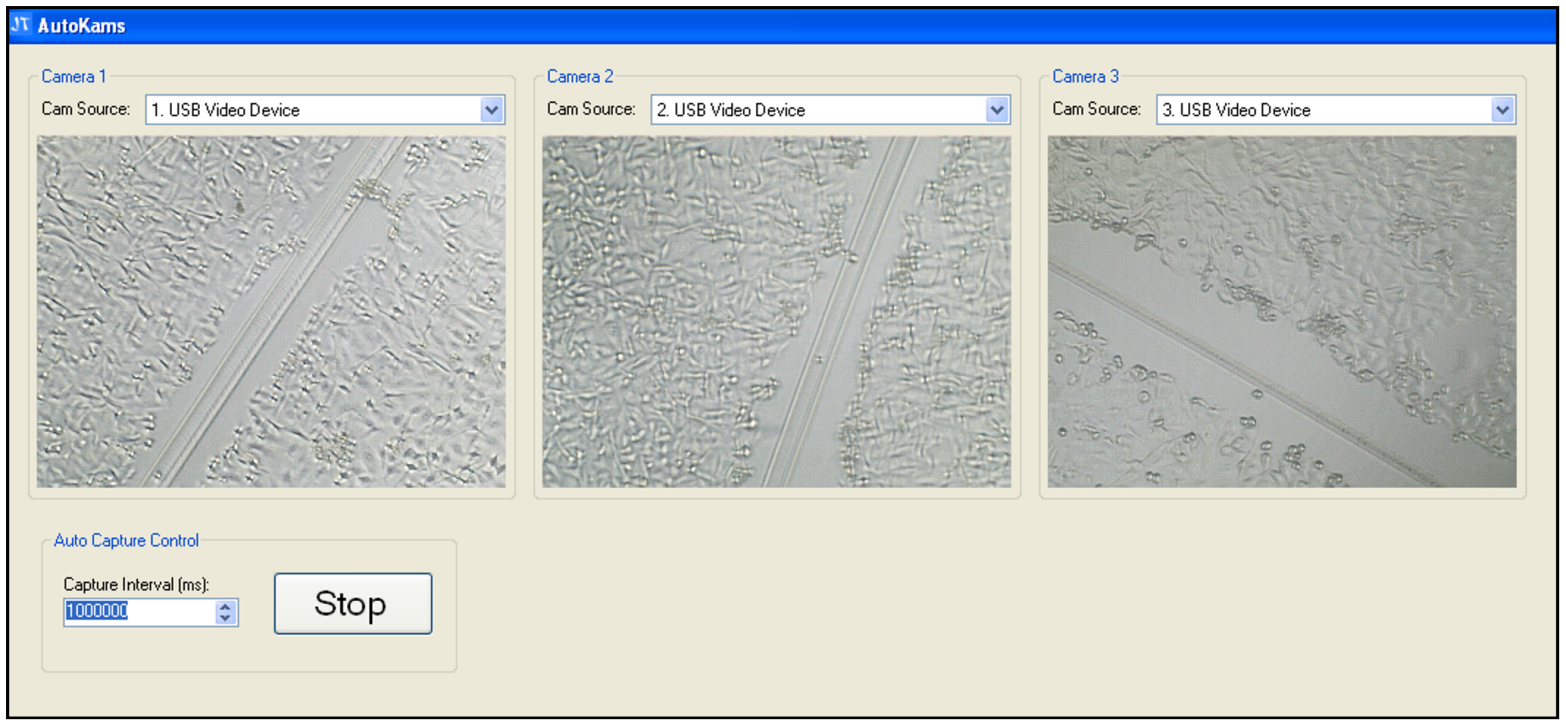
Autokams software interface. Three separate scratch assays of MDA-MB-231 cells displayed simultaneously on Autokams.

The microscopes are bundled with native image capture software, however, due to the unique requirements of this system, it was necessary to write a custom application. ‘AutoKams’ is a standalone application designed to capture images from USB camera devices, such as web cams and microscopes (Figure 2). It was developed using Microsoft Visual Studio 2010 with C# as the programming language. The application is powered by AForge.NET framework (http://www.aforgenet.com/). In many cases when identical USB devices (such as our three microscopes) are connected to the same PC they conflict as the computer is unable to identify them uniquely, this leads to the PC crashing, or the display of only a single camera. Although some devices are able to run in tandem, this was not the case with any of the microscope models we tested. Autokams was written to allow for the simultaneous capture of multiple USB devices of the same model when running on a machine with multiple USB controllers. Images for time-lapse studies were typically captured every 60,000 milliseconds for 1 hour using the capture control box and are automatically saved to labelled folders. Our software is open-source, licensed under LGPL v3, and can be downloaded at: http://lab.junian.net/AutoKams. The software was tested for stability on several Windows PCs, altered accordingly, and was shown to be compatible with three different models of USB microscope (400× maximum magnification, 8 LED, 2 MP interpolated CMOS). The version described here remained stable throughout all our experiments.

#### Image preparation and analysis

**Figure 3:**
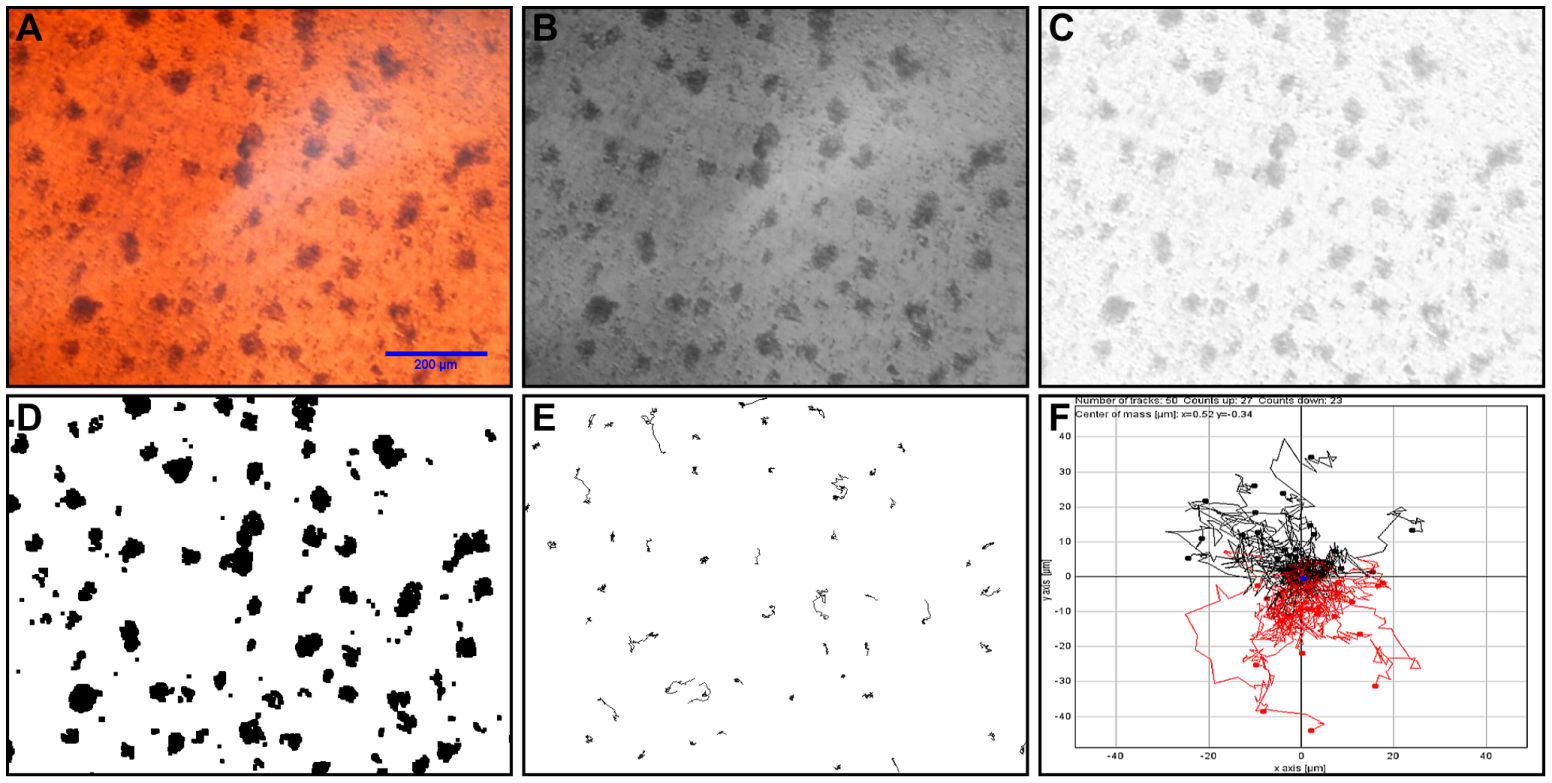
Analysis process in ImageJ. (a) Original image; (b) 8-bit black and white; (c) Background subtraction; (d) Thresholded; (e) MTrack2 cell trajectories; (f) Chemotaxis plot.

Image preparation and analysis was performed within the open-source Java-based image processing program ImageJ (NIH, version 1.44). To set the precise scale an image of an object of known distance was uploaded, then, using the ‘set scale’ option the corresponding size in pixels could be set and applied to other objects. Experimental images (Figure 3A) were loaded as a stack and converted to 8-bit (Figure 3B). Uneven illumination often occurs in microscope images and is difficult to eliminate completely, for this reason the images were also subject to the ‘subtract background’ tool (Figure 3C). The stack was then thresholded to create a binary image with better contrast between cells and background (Figure 3D). The final stage of image preparation was to correct for any minor drift that may occur; this was achieved using the ‘image stabilizer’ plug-in [17]. To analyse cell motility the plug-in ‘MTrack2’ by Nico Stuurman was used (Figure 3E). Minimum track length was set at 10 frames. Although there was no chemoattractant used we also analysed the same data using the ImageJ chemotaxis tool by Gerhard Trapp as an example of further applications (Figure 3F).

### Assays

#### Cell tracking

Cell tracking was performed on cells from three different species: primary hemocytes (immune cells) from the freshwater snail *Biomphalaria glabrata*, mouse juvenile Sertoli cells (SC5) [20] and human breast cancer epithelial cells (MDA-MB-231) [21]. Stock *B.glabrata* were maintained in our laboratory in flow-through tanks at 27°C with a 12 hour light and dark cycle and fed on commercial fish flakes. Sterile *B.glabrata* hemolymph was collected according to Fryer and Bayne (1995) [22] and cells were diluted 50% in snail saline (CBSS PH 7.4; Chernin, 1963 [23]) and allowed to settle for 30 minutes at 27°C before time-lapse began. *B.glabrata* hemocytes were prepared in two separate conditions; cells were either left to attach to the plate or were placed onto a surface of 0.01% poly-L-lysine according to Boehmler *et al.* (1996) [24] with some minor modifications. Both the SC5 and MDA-MB-231 cells were seeded into wells of a 96-well plate in phenol red-free DMEM medium (10% fbs, 1% Glutamax and 1% penicillin-streptomycin - Sigma Aldrich) and allowed to reach approximately 20–30% confluence. The mammalian cells were kept at 37°C during imaging. All time-lapse images were taken every minute for 1 hour.

#### Scratch assay

Scratch assays are a very widely used method to investigate wound healing in various cell types [25]. We performed scratch assays using MDA-MB-231 cells seeded into 3 wells of a 6-well plate to near full confluence using the same culture media as for cell tracking. Scratches were made in each well using the tip of a sterile scalpel. Each well was positioned over a separate microscope, and the thermostat was set to 37°C. Images were captured every 4 minutes from each camera over a period of 10 hours.

#### *Artemia* development

The effectiveness of the microscope for imaging three very different samples simultaneously was tested using shrimp from the genus *Artemia*, as they are a common test organism and developmental stages are clearly distinguishable. Cysts were purchased from ZM foods (Winchester, UK) and were cultured in 20% salinity at 28°C. Samples from different life stages were pipetted into separate wells of a 6-well plate or placed on a microscope slide and imaged at the two different magnifications.

#### Statistical analysis

Analysis was performed on the MTrack2 data generated from the time-lapse files included in supporting information (Movie S1, S2, S3 and S4). As the data failed to meet all the assumptions of an ANOVA and was not amenable to transformation a Kruskal-Wallis test was performed in SPSS version 20 (IMB) to determine whether recorded velocity values differed significantly for the various cell types. As a post-hoc test pairwise comparisons were performed using Dunn's procedure with a Bonferroni correction for multiple comparisons (Figure S4).

## Results

### Microscope Functional Parameters

#### Heating stability

**Figure 4:**
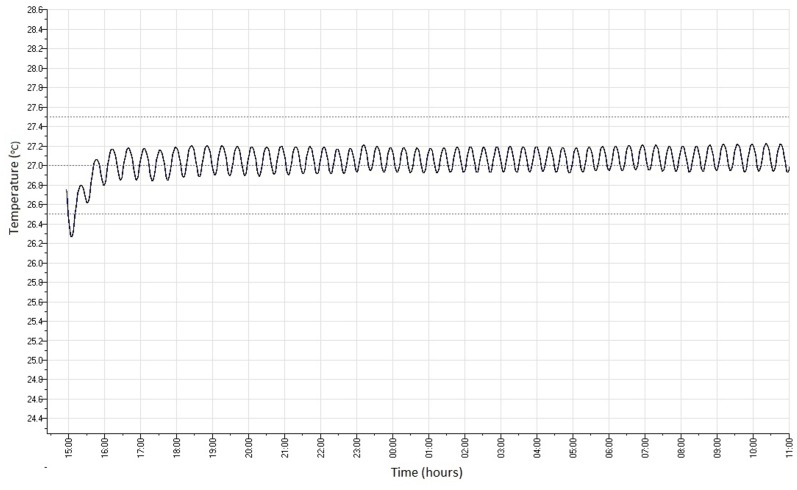
Incubator temperature stability over time. Incubator temperature readings (°C) taken every minute for 20 hours.

After approximately 1 hour the desired temperature (27°C) was achieved (Figure 4). After the first hour the temperature did not vary by more than 0.5°C either side of this temperature for the remaining 19 hours although a gradual increase could be seen.

#### Field of view and magnification

**Figure 5:**
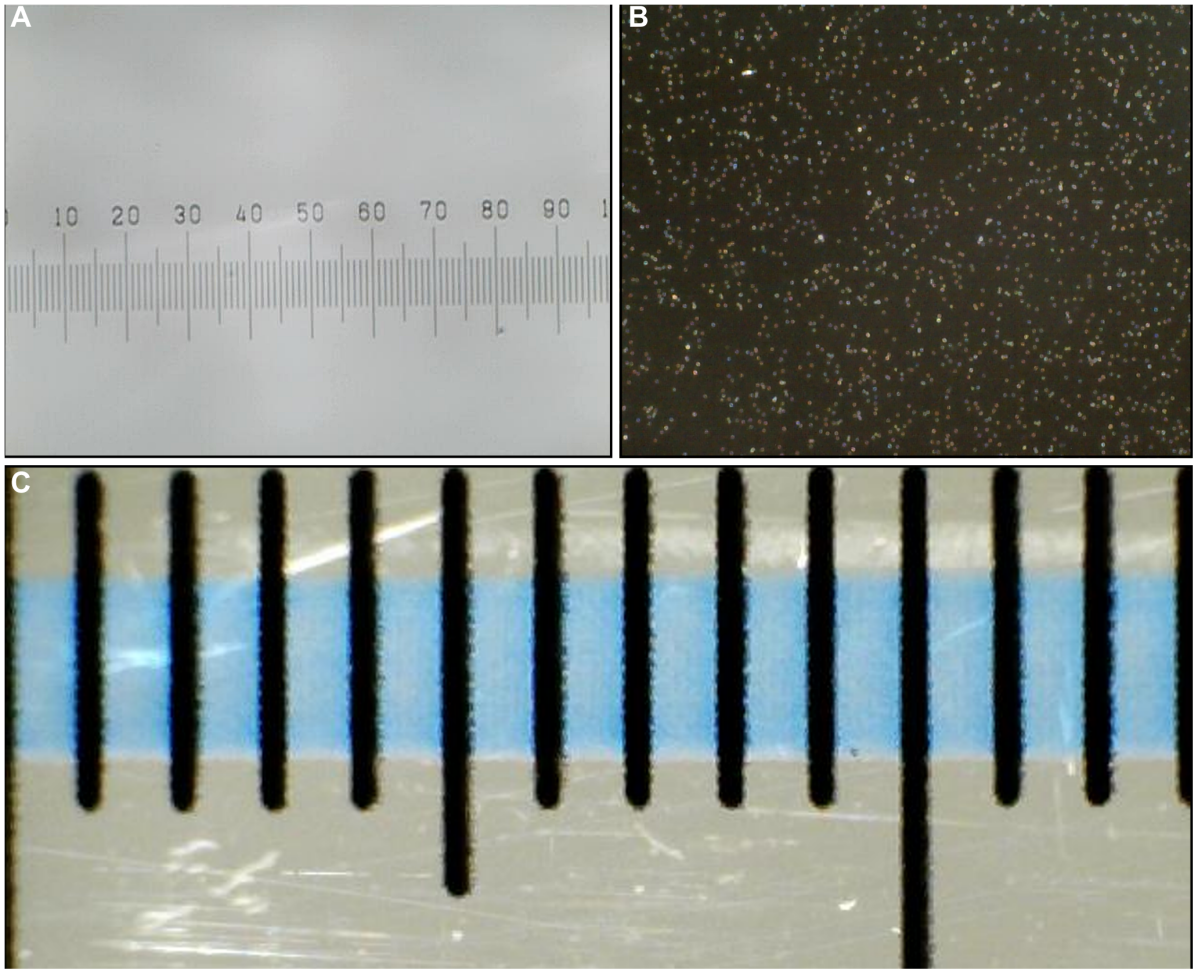
Calibration images. (a) 1 mm graticule with 10 µm subdivisions at 206.8× magnification; (b) 1 µm latex beads at 206.8× magnification; (c) 15 mm ruler at 15.7× magnification with 1 mm subdivisions.

Based on the calibration slide, the horizontal field of view at maximum magnification was determined to be 0.99 mm (Figure 5 A) and 13 mm for the lower magnification (Figure 5 C) at 640×480.

**Figure 6:**
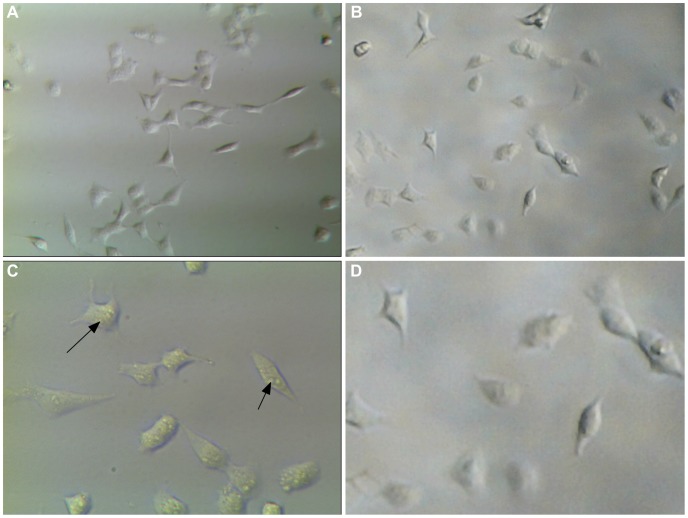
Comparison of MDA-MB-231 cell images between our system and a conventional optical microscope. (a) 310× image taken at 640×480 with a conventional inverted microscope; (b) 206.8× image taken at 640×480 with our system and enlarged post acquisition by 149% to match the size; (c) 620× image taken at 1280×960 with a conventional inverted microscope, arrows show intra-cellular detail; (d) 1280×960 image taken with our system at full magnification (413.6×) and enlarged post-aquisition by 149% to match the size.

We estimated the maximum magnification at the pixel resolution chosen for cell tracking (640×480) to be 206.8× on a screen with a PPI of 78 and the lower magnification to be 15.7× (Figure S3). This calculation appears accurate when we enlarge the image taken with our microscope by 149% (the percentage difference in size between 206.8× and an image taken at the same pixel resolution on a conventional inverted microscope at 310×, (Figure 6 A and B)). The microscopes are capable of greater magnification if pixel resolution is set to the maximum of 1280×960.

#### Pixel resolution

**Figure 7:**
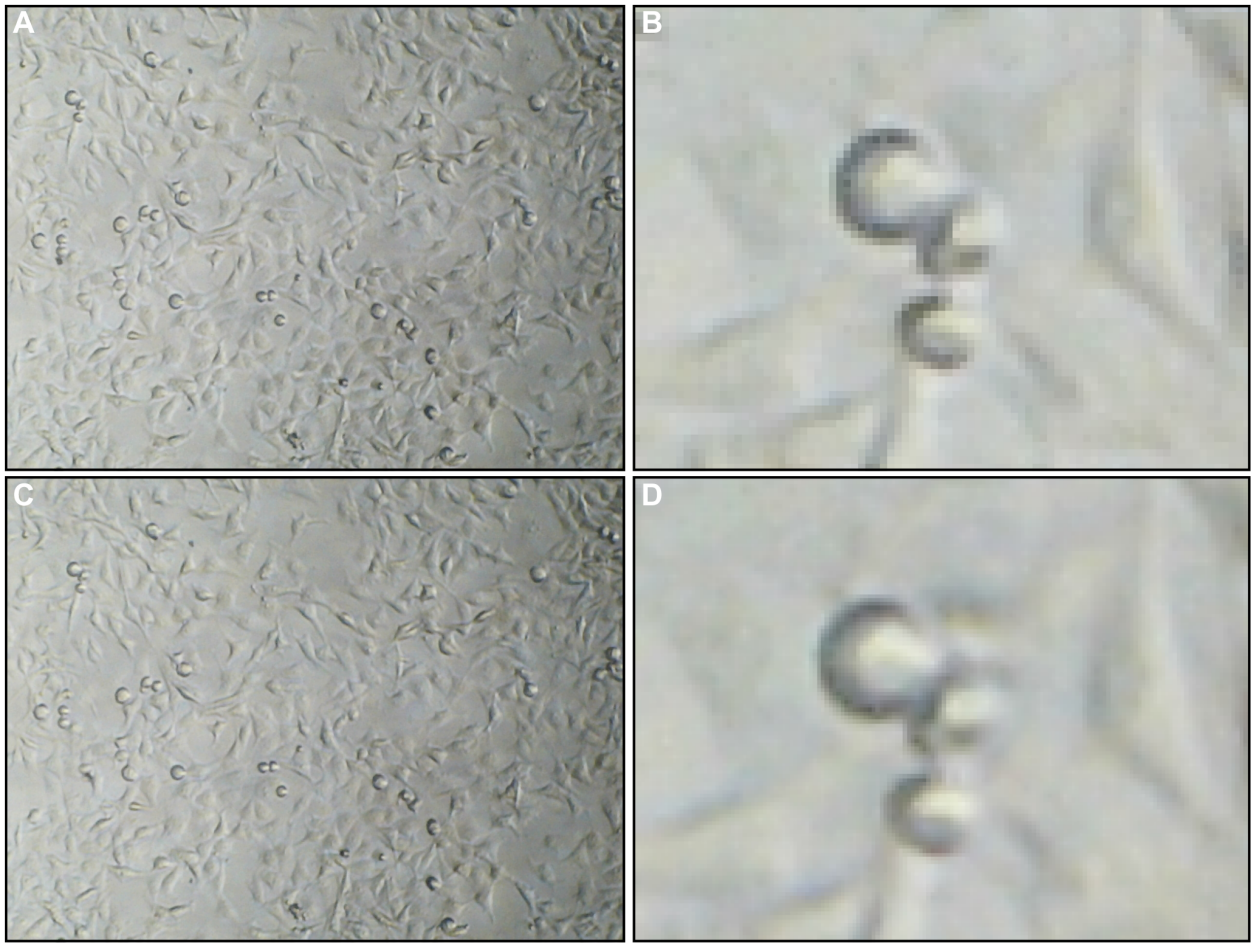
Comparison of digital microscope pixel resolutions between 640×480 and 1280×960. (a) 640×480 image at full magnification; (b) 640×480 image enlarged post-acquisition to 800%; (c) 1280×960 image at full magnification reduced in size by 50%; (d)1280×960 image enlarged post-acquisition to 400%.

The improvement in image quality when increasing pixel resolution from 640×480 to 1280×960 was found to be minimal at the magnification used for cell tracking (Figures 7A and C) but doubles the file size (and therefore storage requirements). The impact only becomes apparent when the images are increased significantly in size post-acquisition, a noticeable increase in pixilation can be seen when enlarging an image taken at 640×480 by 800%, which is the same on-screen size as an image taken at 1280×960 enlarged by 400% (Figures 7B and D), however these sizes are far in excess of what would be useful for our purposes.

#### Spatial resolution

At full magnification, 10 µm divisions were clearly visible and 1 µm beads were distinguishable. However, the measured diameter of the beads was larger than 1 µm suggesting that the microscope was picking up the reflected light (Figure 5B). Spatial resolution was therefore determined to be at least 10 µm. Crucially we were able to clearly resolve all cell types tested (size ranges 24–58 µm) and we could distinguish individual cells from one another and from the background.

#### Comparison with a conventional microscope

The imaging capability of our system was compared to a conventional inverted microscope (GX XDS-3) fitted with a 1.3 mega-pixel camera. The highest magnification on the conventional microscope (620×) was greater than our system (413.6×), maximum pixel resolution of images was the same (1280×960). Spatial resolution on the conventional microscope was higher and intra-cellular detail could be seen at the highest magnification that could not be distinguished in our system when images were enlarged to match the size (Figure 6 C and D). Images can be enlarged post-acquisition, or by using interpolation, to increase on-screen size but this results in ‘empty magnification’ and it resolves no more detail than the original image while resulting in substantial loss of quality (Figure 7 A and B). When comparing the two systems using lower magnification and pixel resolution (as was used for cell tracking assays) we can see little difference in the quality of the images and both would be equally suitable for motility work (Figure 6 A and B). Finally, the cost of assembling our system was approximately £161, whereas a conventional inverted microscope (with camera) retails for approximately £3,250 (Table S1).

#### Lighting

**Figure 8:**
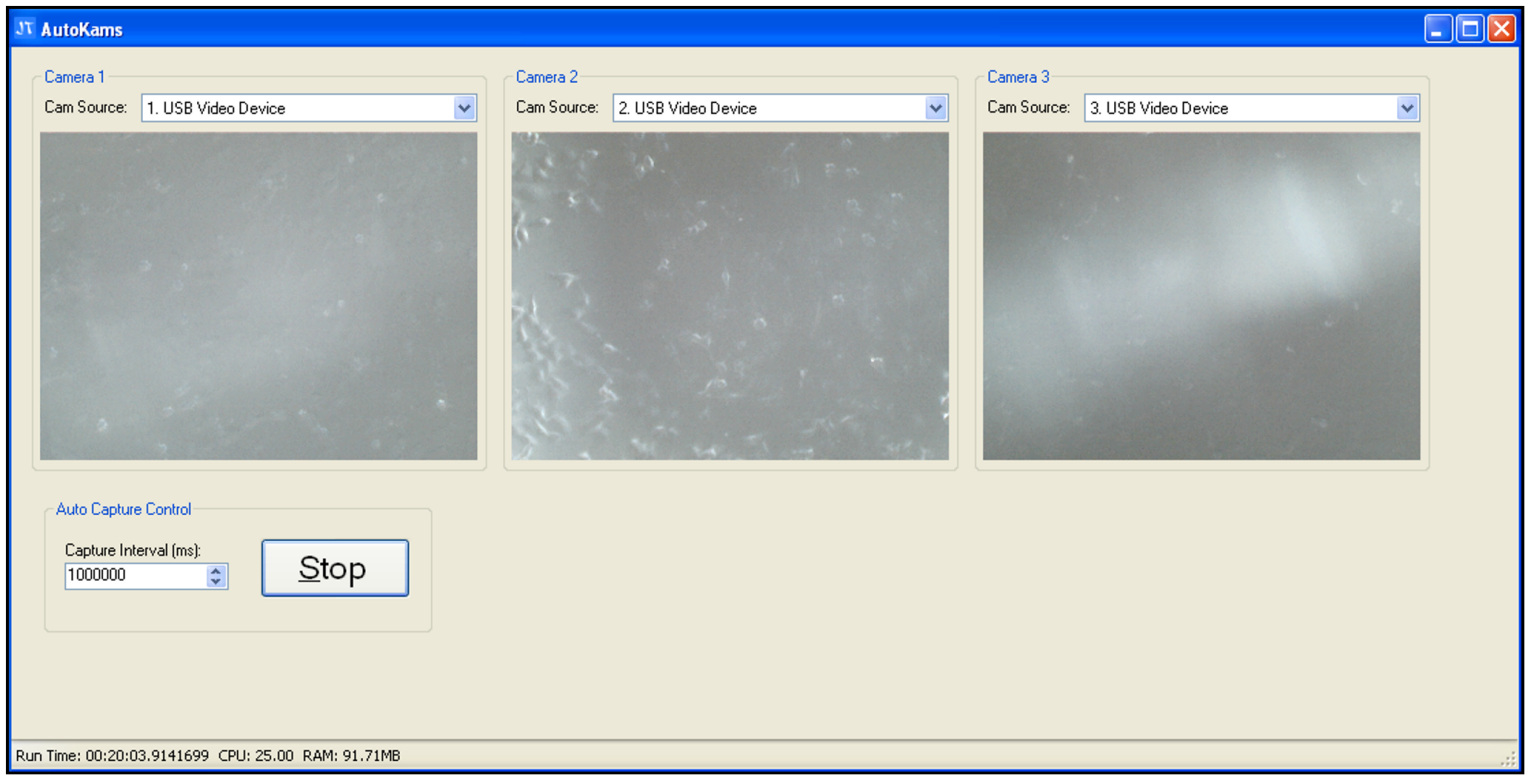
Autokams interface showing the result of illumination from the in-built LEDs alone.

In order to achieve optimum resolution and improve signal-to-noise ratio (SNR) the in-built LED lights were turned off. It was found that for most cell samples optimum SNR was achieved from an external overhead light source (Figure 8).

### Applications

#### Cell velocity

**Figure 9:**
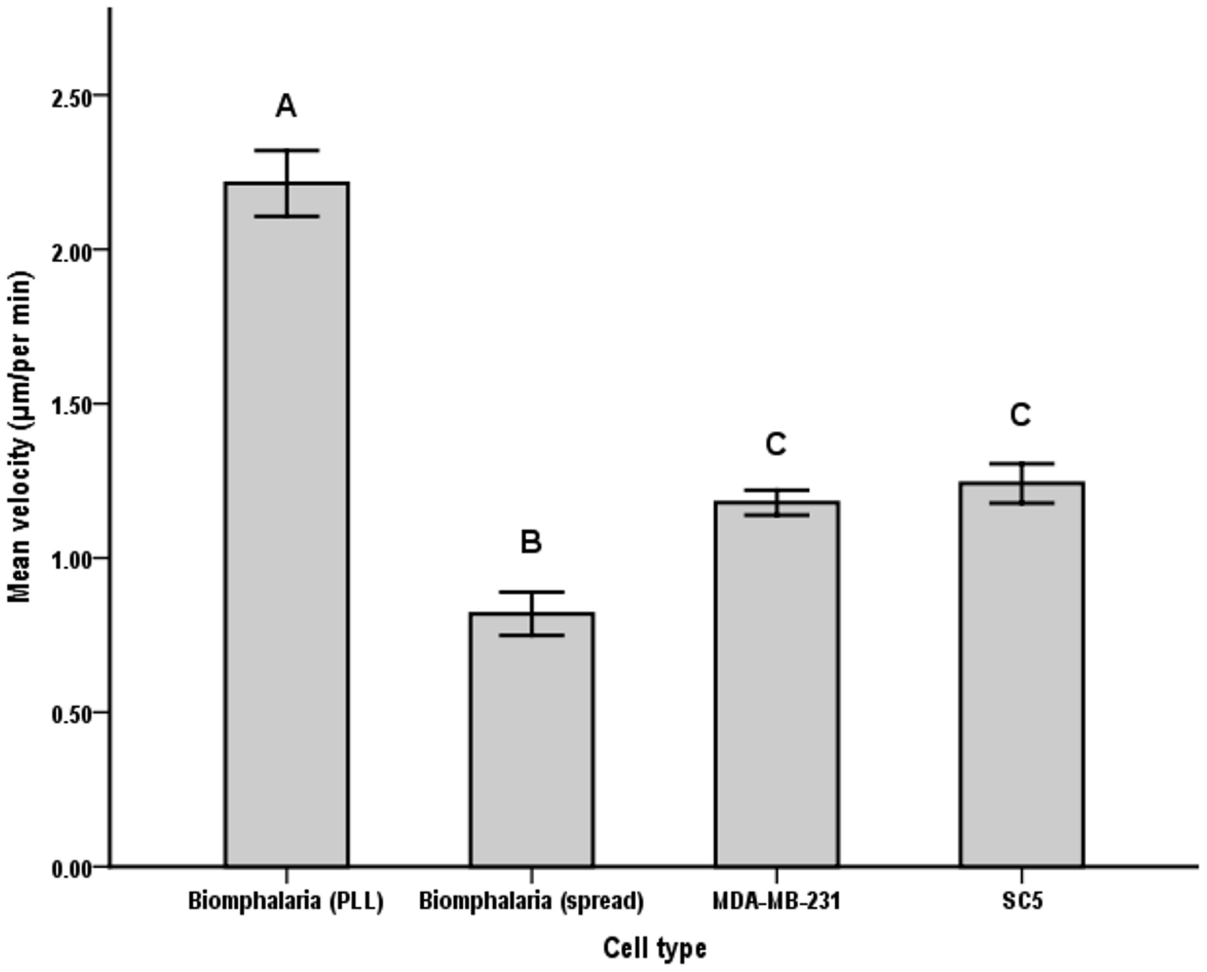
Mean velocity (± SE) in µm per hour of the 4 cell types. Shared letters indicate no significant difference between cell types according to K-W test pairwise comparisons.

According to visual inspection, the Kruskal-Wallis test distributions of velocity scores were not similar for all groups (Figure S4 C), and velocity values differed significantly between cell types, χ^2^ (3) = 106.531, p = >.001 (see Figure S4 A–C). Pairwise comparisons showed significantly lower velocity of spread *B.glabrata* cells (0.81±0.01 µm/min; Figure 9, Movie S4) compared to all other cell types, including *B.glabrata* on PLL (2.21±0.01 µm/min; Figure 9, Movie S1). There were significant differences in velocity between MDA-MB-231 cells (1.17±0.004 µm/min; Figure 9, Movie S2) and both *Biomphalaria* cell culture methods, but not to SC5 (1.24±0.006 µm/min; Figure 9, Movie S3). Likewise, the velocity of SC5 cells differed significantly from both *Biomphlaria* cell culture methods but not from MDA-MB-231 cells (Figure 9). Assumptions of the ANOVA test were most likely not met since we chose to accept results from cells of differing track length (between 10–60 tracks).

#### Scratch Assay

**Figure 10:**
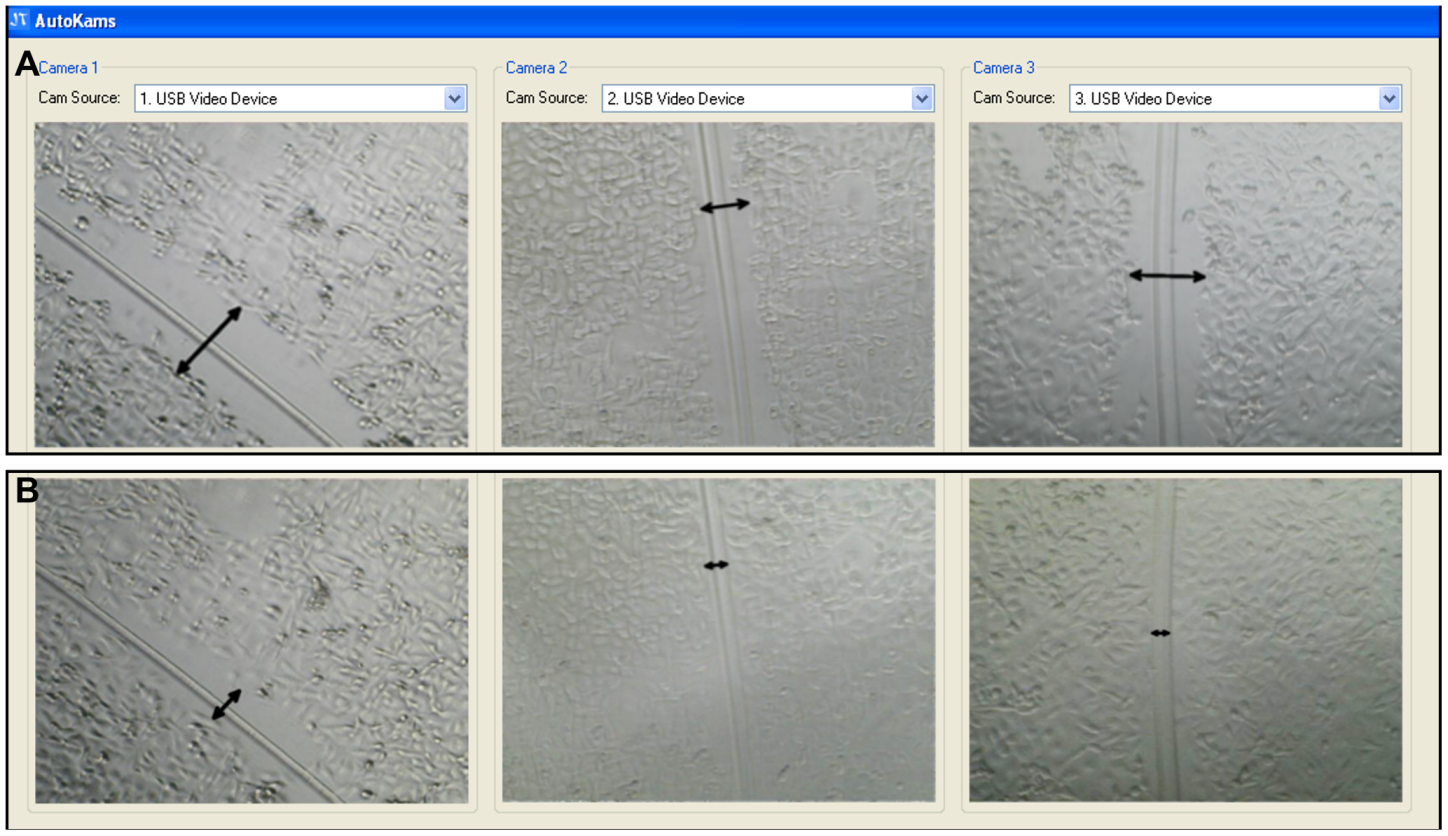
Autokams interface showing individual scratch assays simultaneously recorded on each camera. (a) cells at 0 hr; (b) cells after 10 hrs; Arrows show the scratch channels.

The system was tested for use in performing scratch assays. Not only were the microscopes able to detect cells and scratches sufficient for use in the assay, but it was also found to be relatively simple to align three separate scratches/wells simultaneously (Figure 10; Movies S5, S6 and S7). More difficult was keeping each scratch oriented in the same way, but this has no impact on the validity of the assay and can easily be altered post-acquisition.

#### *Artemia* development

**Figure 11:**
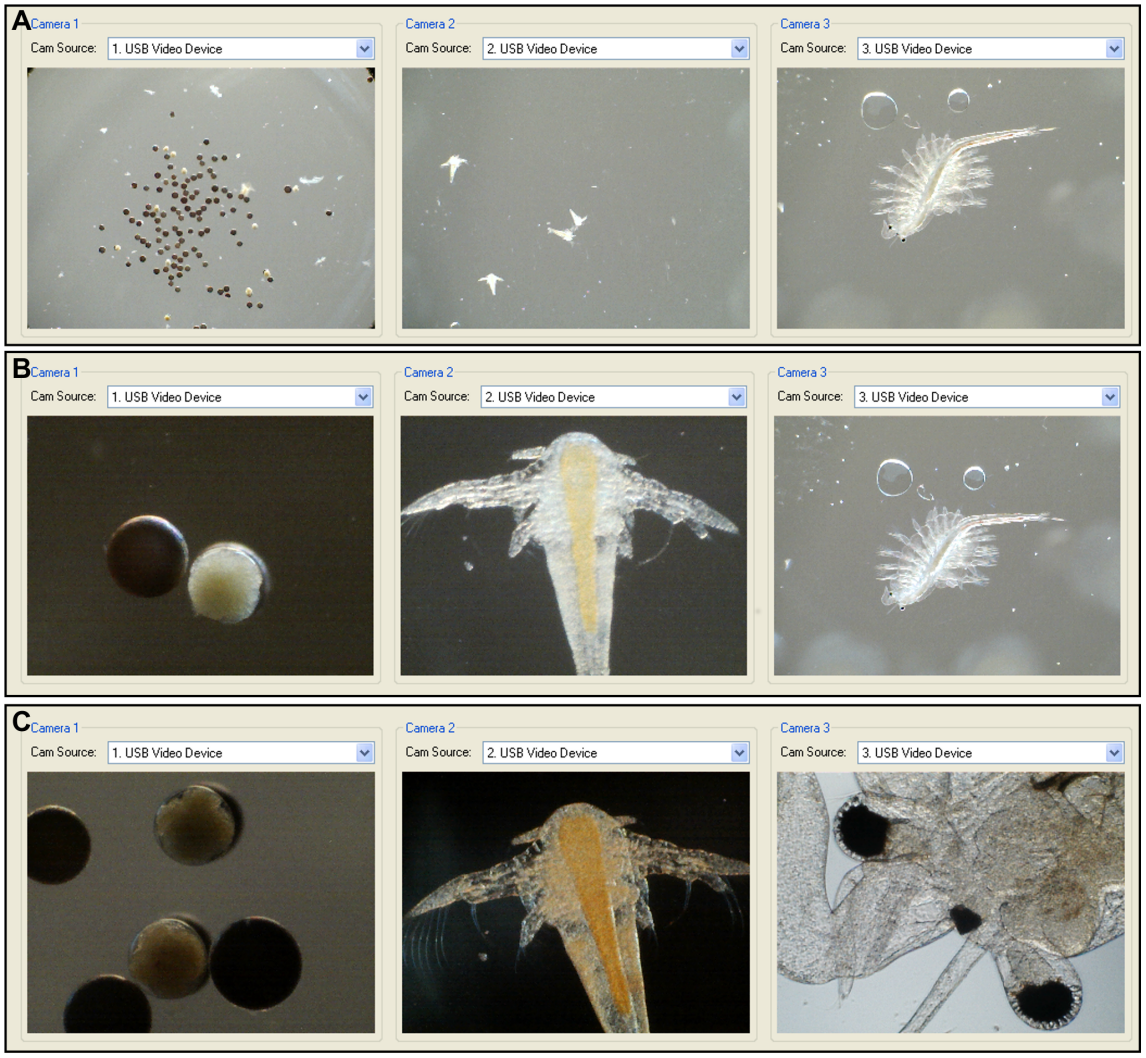
Ability of the microscope system to image distinct samples in different manners simultaneously. (a) Cyst, nauplii and adult *Artemia* at 15.7× magnification; (b) Cyst and nauplii at 206.8×, adult at 15.7×; (c) All three stages at 206.8× magnification.

We were able to demonstrate the ability of the system to simultaneously image three distinct specimens (Figure 11). Furthermore, it was possible to independently alter the magnification of a chosen sample separately. In the case of *Artemia* development, we can view the smaller life stages (cyst, nauplii) in detail by higher magnification while leaving the much larger adult stage at a lower magnification (Figure 11 B).

## Discussion

In this study we demonstrate the development and application of a low-cost microscope system for multiple time-lapse imaging. By inverting commercially available digital microscopes and employing an external light source the stability, working distance and signal-to-noise ratio are improved considerably, and images were comparable to a much more expensive standard inverted microscope/camera system. We also show that the system can support a number of microscopes simultaneously, hence increasing the throughput capability of time-lapse studies captured onto a single PC. Parallel cell tracking and scratch assays were carried out on independent samples with relative simplicity, and the ability of the system to image samples of different sizes simultaneously is demonstrated. There was very little difference in the images taken using our system (at maximum magnification with a pixel resolution of 640×480) compared to a conventional inverted microscope at a similar magnification (Figure 6 A and B). Differences in resolution between the two types of microscope only become apparent when attempting to use higher magnifications (Figure 6 C and D). To achieve the highest useful magnification of the digital microscope (413.6×) the pixel resolution can be set to 1280×960. The maximum magnification of the commercial microscope was 620× (although higher objective lenses can be purchased). Figure 6 C shows an image taken on the conventional microscope at its maximum magnification. As the maximum magnification is higher on the conventional microscope we needed to enlarge our images post-acquisition in order to make a comparison at the same size. Since the limits of the lens and sensor have already been reached for our system this kind of post-acquisition manipulation is an example of empty magnification (Figure 6 D). There is considerably greater detail in the image taken with the conventional microscope due to its greater spatial resolution. Clearly our system would not be suitable for investigating intracellular events, but is perfectly adequate, even at a lower pixel resolution (640×480), for cell tracking. Indeed, greater magnification is not required for this purpose, especially when factoring the increased cost [25].

A number of published studies now demonstrate the rapidly improving capabilities of digital imaging sensors compared to the existing standards in microscopy or explore their potential applications[9], [11]–[14], [26]. Despite this, one of the main limitations to many of the lower-cost time-lapse systems is the rate of data collection or ‘throughput’. Here we demonstrate the impressive capability of CMOS sensors to image cells in tandem as an alternative to the expensive systems on a cost-performance basis. As the sensors used are small and light weight, it is possible to decrease the size and cost of our system substantially by sourcing sensors directly from the manufacturer (rather than buying a commercial system sold as a microscope) as these can cost as little as $1.50 a unit [27]. Stripped back sensors would allow more microscopes to fit into a smaller space (for example in a 3×3 grid pattern) and at much lower-cost. The actual number of microscopes that could be run simultaneously on one PC would depend on the processor speed, memory (RAM/cache) and the number of available USB ports/controllers. However, given the low frame rate required for time-lapse studies it is likely that a larger number could be supported. Indeed by sourcing components directly, we believe that the three camera system described here could be reproduced for as little as 20 GBP. The ability to run three identical microscopes in tandem actually proved to be one of the biggest challenges in developing this system. The microscopes’ native software did not support multiple models and we were unable to find any third-party software that met the criteria. Therefore a new software platform was written specifically for our requirements, demonstrating the importance of collaboration between scientists and programmers; a point that has been stressed by others [2], [28]. We hope that the open-source nature of the software will allow it to be more widely adopted and adapted for use with other platforms and in communities such as ImageJ and Micro manager.

When performing cell tracking or wound healing assays it is common to render the images post-acquisition to enhance the signal (cells) to noise (background and artefact) ratio (SNR), especially when using automated analysis programs. This demonstrates that, beyond a certain point, the overall resolution of the original image has little bearing on the quality of the data [29]. In many cases, it may be unnecessary to invest significant sums of money for high resolution images or high magnification when the same data can be obtained using a less expensive microscope. In the case of cell tracking, high magnification results in a reduced field of view and, consequently, lower sample size and statistical power. Obviously there are conditions where it is crucial to obtain as much information from the image as possible and there is also a minimum level needed to perform tracking assays, especially for slow moving objects. However, in situations where robust motility data is prized above all (attractive images being a secondary consideration) there may be little merit in investing large sums of money.

Four different cell types were used to test the ability of the system to measure cell velocity. *B. glabrata* haemocyte cells moved at a velocity of 0.81 µm/min ±0.01 on an untreated surface, compared to 2.21 µm/min ±0.01 on a poly-L-lysine treated surface (Figure 9). This is consistent with previous reports of an increase in velocity from 0.99 µm/min ±0.72 (untreated) to 5.13 µm/min ±2.02 on poly-L-lysine coated surfaces using *B. glabrata* haemocytes [24], albeit from a different snail strain. For SC5 cells an average velocity of 1.24 µm/min ±0.01 was recorded but we were unable to find sufficient data in the literature for comparison. For MDA-MB-231 cells we showed an average cell velocity of 1.17 µm/min ±0.004. There are several existing studies which have investigated motility in MDA-MB-231 cells which vary from 0.4–2.5 µm/min[30]–[34], likely due to the variation in experimental techniques. Our results are in the middle of this reported range, demonstrating that our system is capable of generating quantitative data comparable to those reported in other studies using more expensive equipment.

While the system we have described cannot match the impressive capabilities of the high-end devices needed for large throughput tasks, such as drug discovery, we believe our system would appeal to researchers with limited budgets. In these circumstances the system could be applied to life science education including demonstrations of different stages of development and the effect of exposure conditions in various macro and microscopic organisms (Figure 11). Our system produces results comparable to standard inverted microscopes, whilst generating three times the data in a single run. The low memory demands of our system mean that it is likely to be compatible with older machines; for example, we were able to run experiments successfully using a 10 year-old PC (IBM ThinkCentre 9210). The system is also portable, enabling experiments to be carried out directly in the field.

There are still many areas in which the system could be improved given more time. While the current manual focusing system works well we believe that this process could be automated by the software in order to reduce the likelihood of accidentally moving the field of view while touching the microscope. An important feature which our system currently lacks, but is commonly required for cell culture, is a CO_2_ pump. The lack of a CO_2_ pump is not believed to have affected the cell tracking experiments shown here, as the DMEM media contains CO_2_, which would not have depleted in the course of 1 hour [35]. The absence of a CO_2_ pump may, however, affect longer-term experiments (such as the scratch assay), and this could be addressed by placing the entire system inside a CO_2_ incubator or finding ways to miniaturize the CO_2_ supply system. One such design, with the inclusion of a miniaturized CO_2_ delivery system, is described by Ho *et al* [36].

## Conclusions

In conclusion, we demonstrate the ability of a novel cell tracking system to perform multiple simultaneous time-lapse studies on various cell types. Due to its low-cost, portability and commercially available components we believe that this system has the potential to enable time-lapse studies by non-specialist departments and schools, and may be a practical solution for researchers with limited financial resources.

## Supporting Information

Figure S1 **Exploded-view.** (a) Microscope supports (b) Base plate (c) Support legs (d) Back piece (e) Thermostat (f) Stage (g) Backboard (h) Incubator (i) Heating cable.
(TIF)

Figure S2 **CAD model of alternative construction plan for microscope housing.** (a) 2D plan of stage and incubator (b) 3D model of assembled unit.
(TIF)

Figure S3 **Magnification equations.** Where ‘DR’ is the diagonal resolution in number of pixels, ‘WP’ is the Width in pixels, ‘HP’ is Height in pixels, ‘PPI’ is pixels per inch for the specific screen, ‘ASP’ is the actual size of a pixel in mm, ‘DS’ is the diagonal size of the screen in inches, ‘M’ is magnification (how many times larger the object on screen is compared to the original), ‘IWP’ is image width in pixels and ‘HFOV’ is the horizontal field of view at the chosen magnification in mm.
(DOCX)

Figure S4 **SPSS output for Kruskal-Wallis test.** (a) Hypothesis test summary (b) Pairwise comparisons of cell type (c) Independent samples box and whisker plot.
(TIF)

Table S1 **Hardware components and prices.**
(DOCX)

Movie S1 **60 minute time-lapse of B.glabrata hemocytes on 0.01% poly-L-lysine surface.** AVI file of image stack created in ImageJ, converted to 8-bit and stabilized.
(AVI)

Movie S2 **60 minute time-lapse of MDA-MB-231 cells.** AVI file of image stack created in ImageJ, converted to 8-bit and stabilized.
(AVI)

Movie S3 **60 minute time-lapse of SC5 mouse Sertoli cells.** AVI file of image stack created in ImageJ, converted to 8-bit and stabilized.
(AVI)

Movie S4 **60 minute time-lapse of B.glabrata hemocytes spread.** AVI file of image stack created in ImageJ, converted to 8-bit and stabilized.
(AVI)

Movie S5 **10 hour scratch assay time-lapse of MDA-MB-231 cells, camera 1.** AVI file of image stack created in ImageJ, converted to 8-bit and stabilized.
(AVI)

Movie S6 **10 hour scratch assay time-lapse of MDA-MB-231 cells, camera 2.** AVI file of image stack created in ImageJ, converted to 8-bit and stabilized.
(AVI)

Movie S7 **10 hour scratch assay time-lapse of MDA-MB-231 cells, camera 3.** AVI file of image stack created in ImageJ, converted to 8-bit and stabilized.
(AVI)

Protocol S1 **Construction method for original microscope system.**
(DOCX)

Protocol S2 **Construction method for alternative design.**
(DOCX)
