# Supplementary figures and images for: Low-Cost Motility Tracking System (LOCOMOTIS) for Time-Lapse Microscopy Applications and Cell Visualisation
Source: PLoS One. 2014 Aug 14;9(8):e103547. doi: 10.1371/journal.pone.0103547 (PMC4133191; doi:10.1371/journal.pone.0103547)

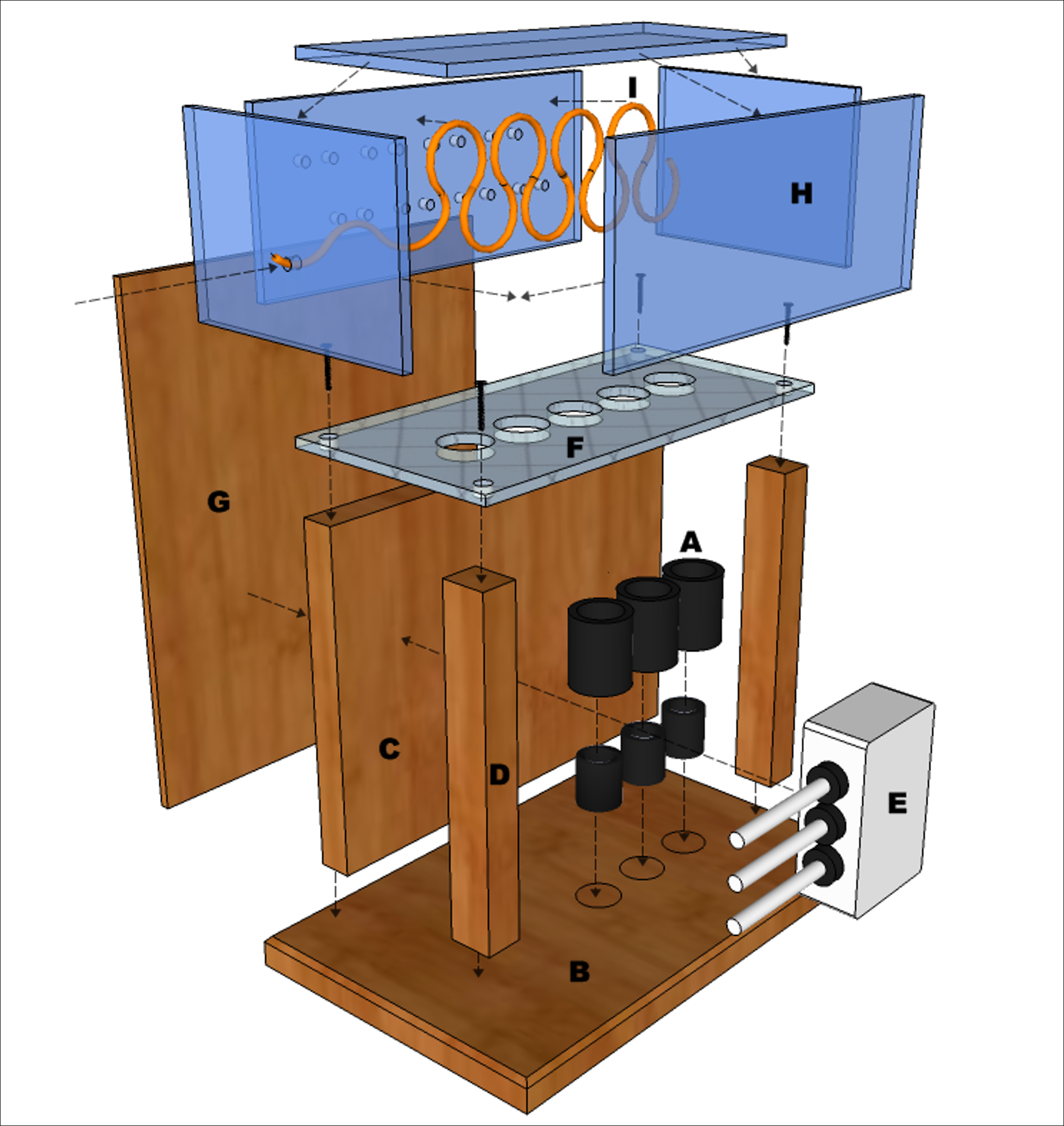

Supplement: Figure S1 — Exploded-view. (a) Microscope supports (b) Base plate (c) Support legs (d) Back piece (e) Thermostat (f) Stage (g) Backboard (h) Incubator (i) Heating cable. (TIF) [file pone.0103547.s001.tif]

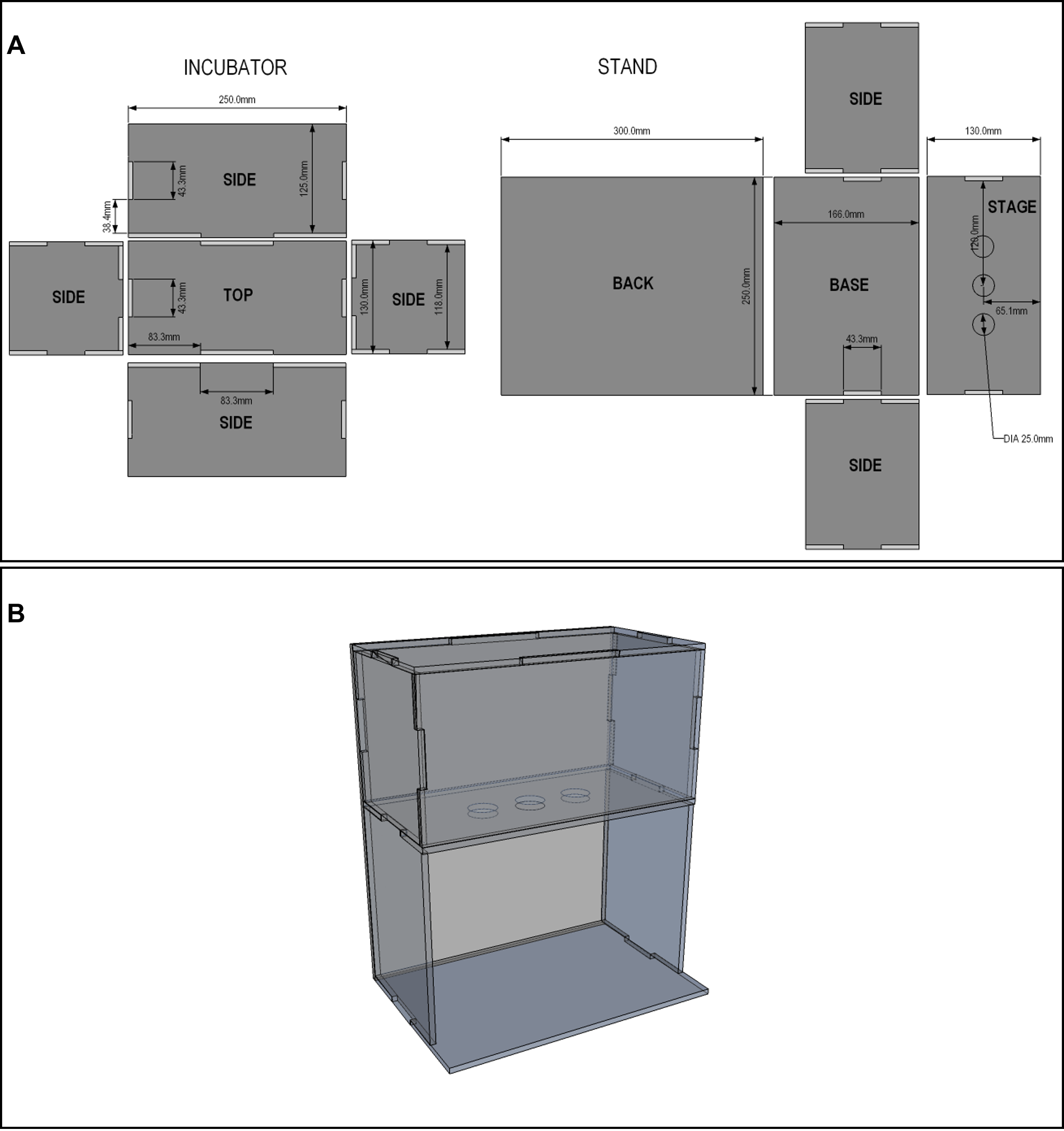

Supplement: Figure S2 — CAD model of alternative construction plan for microscope housing. (a) 2D plan of stage and incubator (b) 3D model of assembled unit. (TIF) [file pone.0103547.s002.tif]

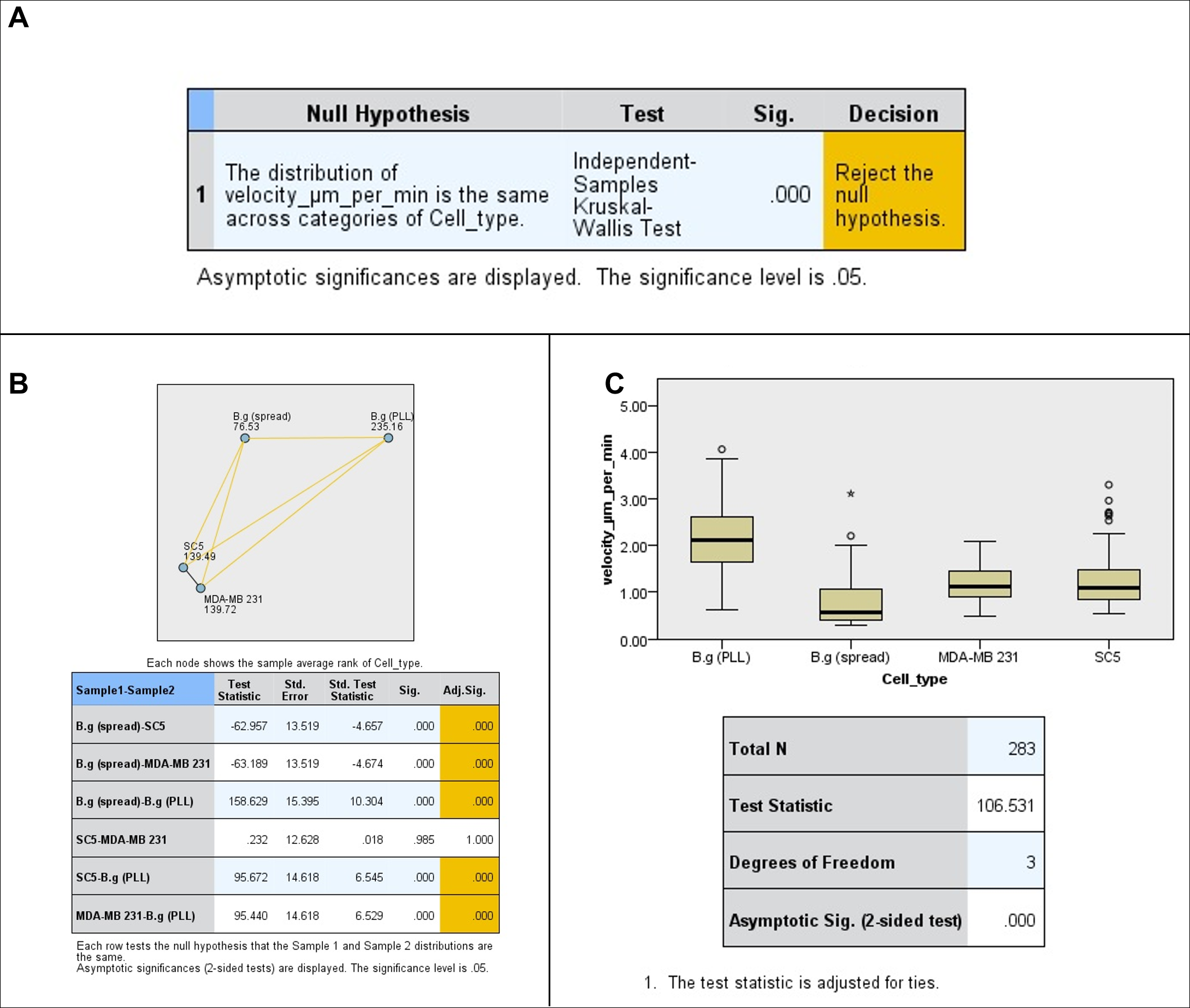

Supplement: Figure S4 — SPSS output for Kruskal-Wallis test. (a) Hypothesis test summary (b) Pairwise comparisons of cell type (c) Independent samples box and whisker plot. (TIF) [file pone.0103547.s004.tif]
