# Supplementary material for: Low-Cost Motility Tracking System (LOCOMOTIS) for Time-Lapse Microscopy Applications and Cell Visualisation
Source: PLoS One. 2014 Aug 14;9(8):e103547. doi: 10.1371/journal.pone.0103547 (PMC4133191; doi:10.1371/journal.pone.0103547)
Supplement: Table S1 — Hardware components and prices. (DOCX) [file pone.0103547.s005.docx]

| **Item** | **Details** | **Lowest price** | **Notes** |
| --- | --- | --- | --- |
| Microscope x 3 | Veho VMS-004D – 400x | £90 (£30 per unit) | Retails on website at £69 but can be sourced for significantly less |
| Thermostat | Microclimate, Ministat 100 | £23 | Several models may be suitable including aquarium thermostats |
| Heating cable | Soil warming cable, 3M, 37.5W | £15 | Other heating elements may be suitable including heat mats |
| Incubator | Clear acrylic, 6mm thick | *£1 | Scrap, approx £3 for A4 size (210 × 297 mm) |
| Stand | Oak | *£1 | Scrap |
| Microscope supports x 4 | Kitchen unit legs, pack of 4, 150mm length | £1 | These were cut to size |
| 3.6w LED strip lamp | Flexible | £20 | Other types of LED may be equally suitable |
| Timer plug |  | £10 | Minimum of 1 second on/off intervals |
| Reusable cable tie x2 | 200mm length, £2 for pack of 10 | £0.4 | Optional. Ordinary cable ties can be used |
| Acrylic cement | Weld-on #16, 5 oz. tube | £5 | Other brand of acrylic glue would likely be suitable |
| Draft excluder tape | Self adhesive, 5mm thick, 2x 5M roles | £1.50 | Optional |
|  |  | Total: £161 |  |

*Approximate value where scrap items were used
