# Supplementary material for: Low-Cost Motility Tracking System (LOCOMOTIS) for Time-Lapse Microscopy Applications and Cell Visualisation
Source: PLoS One. 2014 Aug 14;9(8):e103547. doi: 10.1371/journal.pone.0103547 (PMC4133191; doi:10.1371/journal.pone.0103547)
Supplement: Protocol S1 — Construction method for original microscope system. (DOCX) [file pone.0103547.s013.docx]

**Construction Method - See Figure S1**

**Component A: microscope supports**. Adapt the plastic kitchen unit legs by cutting off the base feet and cutting to size the threaded stem and barrel. Slightly chamfer the inside of the barrel to accommodate the bottom of the USB microscope body.

**Component B: base plate**. Using dense plywood, hardwood or MDF, cut the base to size. Mark out the centres of the microscopes and drill holes according to the diameter of the threaded stems (A) to be inserted. Glue the stems into the base using epoxy type glue making sure they are vertical.

**Component C** & **D: support legs & back piece**. Using plywood or hardwood cut these to size to fit the length of the base and the height of the microscopes. Glue C and D to B (standard woodworking glue is sufficient).

**Component E: thermostat**. Position the thermostat on the back board and mark holes for cable ties. Attach the thermostat to C with the cable ties and trim off tie ends.

**Component F: stage**. Mark out the dimensions of the acrylic sheet to suit so it attaches to the back and supports (can have slight overhang to the front and sides if desired). Using a Forstner bit 0.5 - 1.0mm less than the diameter of the microscope lens drill through the acrylic sheet to match the centres of the inserts and stems in the base. Using a round hand file, taper the insides of the scope holes so that the lens is a tight fit and flush with the stage.

**Component G: back board**. Using plywood cut out the back board (G) attach to C using screws or glue.

**Component H & I: incubator & heating cable.** Mark out and cut the acrylic sheet to sizes necessary to house the heating element and so it fits onto and covers the microscope top plate. Glue the sides, top and ends together using acrylic cement and hold in place with ‘quick clamps’ (acrylic cement will set in 20 - 30 sec). Drill a small hole into the side of the incubator and thread the heating cable (I) through. Drill holes in the back of the incubator (H) and secure the heating cable to the inside using cable ties, cut off excess and seal the holes with acrylic glue. Seal all cable tie holes with mastic or foam rubber.

**Tools**

- Hobbyist table band saw (alternatively a hand saw can be used)
- Table pillar drill (alternatively a hand held drill can be used)
- 4 x adjustable ‘quick clamp’ type clamps (other clamps would likely be suitable)
- Forstner drill bit for cutting scope holes in top and baseplate
- Small drill bits for various holes
- Small round hand file to taper the scope lens holes (alternatively sand paper can be used)
- Medium sand paper to smooth cut edges
- Hand scriber to mark wood and acrylic
