# Supplementary material for: Low-Cost Motility Tracking System (LOCOMOTIS) for Time-Lapse Microscopy Applications and Cell Visualisation
Source: PLoS One. 2014 Aug 14;9(8):e103547. doi: 10.1371/journal.pone.0103547 (PMC4133191; doi:10.1371/journal.pone.0103547)
Supplement: Protocol S2 — Construction method for alternative design. (DOCX) [file pone.0103547.s014.docx]

**Construction method – See Figure S2**

**1.**Mark out the sides, tops and bases onto the acrylic sheet using a hand scriber according to Figure S2 (a).
**2.**Cut out all components using a band saw with guide set to suit (allow for blade width of cut ). Lugs (lighter gray area, figure S2 (a)) can be cut out for increased stability or the sides can be left square for more simple construction.
**3.** Drill out the holes marked on the stage for the microscope lens’ using a Forstner bit (drill right through). Using a round hand file taper the inside of the scope holes until the lens fits secure and flush with the top of the stage.
**4.** Adapt the kitchen unit legs: cut off the plastic foot and cut the threaded stem and barrel to size. Cut out slots in the threaded stems (the bottom/thinner part) to feed the USB cables through.
**5.** Cement the threaded stems into the base plate ensuring that they are vertical (acrylic cement will set in 20 - 30 seconds).
**6.** Cement the ends of the box to the back using the 4 x clamps, check they are vertical and remove excess cement.
**7.** Cement the scope base to the above
**8.** Repeat for the stage.
**9.** Repeat the process for the incubator box.
**10.** Drill and fix the heating element using cable ties as for the original microscope (Figure S1, Protocol S1)
**11.** Secure all cables using cable ties (if required the assembly can be fixed to a wooden or MDF base / work surface). Draft excluder tape can be used on the bottom of the incubator box to ensure it is sealed.
