# Supplementary material for: Low-Cost Motility Tracking System (LOCOMOTIS) for Time-Lapse Microscopy Applications and Cell Visualisation
Source: PLoS One. 2014 Aug 14;9(8):e103547. doi: 10.1371/journal.pone.0103547 (PMC4133191; doi:10.1371/journal.pone.0103547)
Supplement: Supplementary file 5 [file pone.0103547.s003.docx]

$$DR = \sqrt{WP^2 + HP^2}$$

$$PPI = \frac{DR}{DS(inches)}$$

$$ASP(mm) = \frac{25.4}{PPI}$$

$$M = \frac{IWP \times ASP(mm)}{HFOV(mm)}$$
